# Upgrading Data Sharing Policies to Maximize Utility and Impact in Genetics and Genomics Research

**DOI:** 10.1002/ggn2.202400055

**Published:** 2024-12-27

**Authors:** Yuming Hu, Lei Lei, Kerstin Brachhold

Sharing one's evidence has been at the core of what we now call “science” for hundreds of years. Roger Bacon wrote in the 13th century that “theories supplied by reason should be verified by sensory data, aided by instruments, and corroborated by trustworthy witnesses.”^[^ ^1^ ^]^ In the modern age, when all science is aided by computers and rich troves of digital data, is it not entirely normal for us, as trustworthy witnesses, to expect to see these data before we accede to someone else's conclusions? Gregor Mendel's transformative paper on pea genetics from 1866 is full of tables sharing “raw” data; tables which themselves have led to years of healthy statistical debate regarding whether Mendel or his assistants may have artificially cleaned their data to produce more idealized outcomes.^[^ ^2^ ^]^

In this light, modern data availability debates should not be considered something new, but instead another step in an ongoing movement that aims to build a shared and empirically grounded understanding of our natural world.

This month, *Advanced Genetics* is joining other Wiley journals in implementing a “Mandates Data Sharing” policy (Data Sharing Policy | Wiley) (https://authorservices.wiley.com/author‐resources/Journal‐Authors/open-access/data‐sharing‐citation/data‐sharing‐policy.html). We and the other participating journals require that authors openly share data underlying their publications and upgrade our editorial workflows to better support data sharing and optional data peer‐review. This initiative covers 88 Wiley Journals by now across various fields, such as cell and molecular biology, genetics, geoscience, microbiology, plant science, physics, computer science, and social science (Table S1, Supporting Information). We also intend to mandate minimum standards for that data, as per an initiative that began in 2023 with a group of Wiley Ecology & Evolution Journals.^[^ ^3^ ^]^ It aligns with the focus on open science and open data sharing from major science funders around the world. The declaration from the White House Office of Science and Technology Policy in 2022, which strengths data sharing requirements for US‐funded researchers, is only one example of this growing trend (https://www.whitehouse.gov/ostp/news‐updates/2022/08/25/breakthroughs‐for‐alldelivering‐equitable‐access‐to‐americas‐research/).

Open sharing of nucleotide sequence data through the interlinked databases of the International Nucleotide Sequence Database Collaboration (https://www.insdc.org/) has been a standard condition of publication at major genetics journals for over three decades, and many genetics and molecular biology journals require that authors similarly deposit and share other “omics” data‐types, for which the community has developed a range of high‐quality centralized public repositories such as GenBank (https://www.ncbi.nlm.nih.gov/genbank/), ArrayExpress (https://www.ebi.ac.uk/arrayexpress/), Genomic Expression Archive (https://www.ddbj.nig.ac.jp/gea/index‐e.html), Genome Sequence Archive (https://ngdc.cncb.ac.cn/gsa/).

For datasets that do not have a dedicated repository, authors will be encouraged to deposit data to an appropriate generalist data repository, such as Dryad (https://datadryad.org/), Figshare (https://figshare.com), OSF (https://osf.io), or a suitable DOI‐issuing institutional data repository. Authors are encouraged to fully deposit their data before they submit their manuscript. Many repositories allow authors to keep data private until final publication, and, in these cases, authors should include links or login details for reviewers in their submissions. Similarly, software source code, mathematical models, and other digital research objects should be shared in appropriate repositories, such as Zenodo (https://zenodo.org/), whenever possible.

Those in search of a suitable data repository are encouraged to browse the options available at FAIRsharing (https://fairsharing.org/). This website also includes useful information on community‐developed reporting standards, which can help authors share data in a manner that maximizes its reusability.^[^ ^4^ ^]^

For human subject studies, all submitted data must be de‐identified. For sensitive human data that cannot be openly shared, we encourage authors to provide clear information on how other researchers may request the data, including links to data use agreements when relevant. Whenever possible, sensitive data should be shared through community‐maintained controlled‐access repositories. In the genetics field, excellent choices include the European Genome‐phenome Archive (EGA, https://ega‐archive.org/) and the Database of Genotypes and Phenotypes (dbGaP) (https://www.ncbi.nlm.nih.gov/gap/). If any data cannot be shared, this should be clearly stated with a brief explanation. Limitations on data sharing for reasons other than human privacy will be considered on a case‐by‐case basis by the editors of the journal and should be clearly justified in each submission.

For more information on these new data‐sharing policies, and more advice on sharing data, please refer to this Academic Practice article at *Ecology and Evolution*.^[^ ^3^ ^]^ We hope our authors will fully embrace our endeavor to make science more open and accessible.

## Conflict of Interest

The authors declare no conflict of interest.

## Supporting information



Supporting Information
